# Relationships Among Hearing, Auditory Processing, and Cognition: A Study of Older Adults

**DOI:** 10.3390/audiolres16050131

**Published:** 2026-09-03

**Authors:** Kristina Mullins, Jennifer Jones Lister, Aryn L. Harrison Bush, Laura Conover, Nasreen Sadeq

**Affiliations:** 1 Department of Communication Sciences and Disorders, University of South Florida, Tampa, FL 33620, USA; kris.mullins29@gmail.com (K.M.); aryn@usf.edu (A.L.H.B.); lconover1@usf.edu (L.C.); 2 School of Aging Studies, University of South Florida, Tampa, FL 33620, USA; nsadeq@usf.edu

**Keywords:** cognitive aging, hearing loss, auditory processing, binaural processing, dichotic listening, CogState, MoCA, DSI

## Abstract

**Background/Objectives**: Beginning in mid-life, age-related cognitive decline may negatively affect overall hearing health. Thus, understanding the relationship between hearing and cognition is of critical importance. Although the relationship has received considerable attention, and numerous theories have been posited to explain it, questions remain. Research indicates that 50% of older Americans grapple with severe hearing loss, affecting their daily communication, while Alzheimer’s disease, a prevailing cause of dementia, affects approximately 11% of Americans aged 65 or older. The Lancet Commission underscores hearing loss as a leading modifiable risk factor for dementia, contributing up to 7% of the risk. The purpose of this study was to advance our understanding of the specific domains of hearing and cognition that are related, thus informing theory and clinical practice. **Methods**: Participants were 99 older adults (mean age = 73 years) recruited from the 10-year Indicators of Cognitive Change study. Measures of peripheral hearing sensitivity (four-frequency pure-tone average [PTA4]) and auditory processing (Dichotic Sentence Identification [DSI]) were used to predict performance on multiple measures from the Montreal Cognitive Assessment (MoCA) and Cogstate Brief Battery (CBB). **Results**: DSI emerged as a significant predictor across both MoCA and CBB, surpassing the predictive power of PTA4. Surprisingly, relationships between hearing and cognitive measures differed for MoCA domains versus CBB subtests, highlighting the complexity of this association. **Conclusions**: The ability to separate and identify competing auditory information may be necessary components of healthy cognitive function. In clinical practice, assessment of binaural auditory processing may provide an early indicator of cognitive vulnerability in older adults.

## 1. Introduction

Hearing loss and cognitive impairment are common among older adults; thus, audiology patients may present with varying degrees of both disorders which will interact to influence hearing healthcare outcomes. In the United States of America (USA), approximately 50% of older adults between the ages of 60 to 69 have hearing loss that is so severe that it affects their daily communication [1]. Worldwide, 58% of adults aged 60 and older have moderate or worse hearing loss [2]. Approximately 11% of older Americans 65 and older have been diagnosed with Alzheimer’s disease (AD) [3], the most common cause of dementia; when factoring in other causes of dementia, the rate increases up to 14–15% [3]. An estimated 57 million people worldwide are struggling with dementia [4]. According to the Lancet Commission [5], hearing loss is one of the top potentially modifiable mid-life risk factors for dementia, accounting for up to 7% of risk. However, the field of audiology has struggled to determine how best to incorporate information on the relationship between hearing and cognition into clinical practice, partially because the mechanisms underlying the relationship are not entirely clear [6,7,8].

It is important to note that both hearing loss and cognitive impairment carry a heavy financial and emotional cost. Over a two-year period, older Americans with hearing loss incurred 26% higher healthcare costs than those without hearing loss. These cost differences rose to 37% at five years and 47% at ten years [9]. Across similar time frames, hearing loss was associated with greater risk of dementia (~50%) and depression (~40%), as well as fractures, falls, acute myocardial infarction, and stroke [10]. Additionally, older Americans with hearing loss experience hospitalization, death, and frailty at higher rates compared to their normal-hearing counterparts [1].

It is estimated that dementia will cost the USA $409 billion in 2026 and up to $1 trillion in 2050 [3]. Approximately 12 million people are informal caregivers for people with AD and related dementias (ADRD) and provide nearly 189 billion hours of unpaid care to their loved ones [3]. Evidence suggests that being an informal caregiver may lead to feelings of a meaningless existence, burden, anxiety, stress, and fatigue, which are all risk factors for premature death [11]. Additionally, there is significant fear surrounding dementia in the older adult community [12]. The financial and emotional burden of hearing loss and dementia on patients and their caregivers is tremendous, making early referral, detection, and treatment of critical world-wide importance. Recently, the Alzheimer’s Association reported [13] that 79% of people would want to know if they had ADRD if that knowledge could allow for earlier intervention. This supports the idea that people would be open to evidence-based strategies and appropriate treatment. Audiologists and other allied health professionals—who comprise approximately 60% of the USA. healthcare workforce—are well-positioned to promote cognitive and auditory wellness [14]. By identifying at-risk patients during routine visits, they can facilitate timely referrals to cognitive healthcare specialists. Integrating audiologists into multi-disciplinary healthcare teams can optimize early detection and referral for potential cognitive impairment, ultimately streamlining accurate diagnosis and targeted treatments.

Although epidemiological research has established that poor peripheral hearing (as measured by pure-tone hearing thresholds) is associated with impaired cognition [15,16], the strength of the relationship between peripheral hearing and cognitive performance is weak [17]. Cross-sectional studies rarely show significant differences in peripheral hearing or function of peripheral hearing structures among those with and without cognitive impairment [18,19]. In fact, both cross-sectional and longitudinal studies indicate that auditory processing may be more strongly related to cognition than peripheral hearing [20,21,22,23]. A major limitation of the current body of literature is the limited and mixed array of cognitive and auditory domains included.

Harrison Bush et al. [17] assessed the relationship between pure-tone hearing (three-frequency pure-tone average [PTA] in the better hearing ear) and three domains of cognition (processing speed, executive function, memory). They demonstrated that, after accounting for known risk factors of hearing loss and cognitive impairment, peripheral hearing sensitivity accounted for only a minimal (albeit significant) amount of variance across measures of speed of processing (0.6% to 1%), executive function (0.5% and 1.7%), memory (0.4% to 2.2%), and global cognitive status (0.9%). Results (percentages) were in line with those of previous studies [24]. Nixon et al. [25] found that poorer PTAs (PTA4 in the better hearing ear) predicted poorer performance on the executive function portion of the CogState Brief Battery (CBB; *r* = 0.228, *p* = 0.037). In their 2018 systematic review, Loughrey and colleagues [26] showed that most studies of hearing and cognition included limited and varied cognitive domain measures, highlighting the limitations of this body of work. The present study, therefore, includes two tests of cognition (Montreal Cognitive Assessment [MoCA], CBB) with multiple sub-tests targeting a variety of cognitive domains.

In terms of higher-level auditory processing abilities, the literature is also mixed. A systematic review [27] of 126 published articles on the relationship between auditory processing and cognition showed that auditory processing was most often measured via sentence recognition in noise while cognitive ability was most often measured via working memory. Across studies, the authors were unable to conclusively identify consistent relationships between measures of auditory processing and cognition.

O’Brien et al. [28] used factor analysis to assess auditory processing measures and cognitive measures in adults 55 years and older with (*n* = 73) and without (*n* = 140) probable mild cognitive impairment (MCI). Findings suggested that binaural processing (as measured using the Dichotic Sentence Identification [DSI] test) and cognitive measures tap similar domains whereas temporal processing and measures of degraded speech understanding reflected distinct, separate domains. Notably, binaural measures shared a common factor with measures of executive function and visual speed of processing, including those that measure divided attention. Correlations of binaural measures with cognitive measures were small to moderate in effect size. Most recently, Lister et al. [29] demonstrated significant differences between older adults with and without a clinical diagnosis of MCI on measures of binaural processing (*p*s ≤ 0.006), but not on measures of temporal processing or degraded speech (*p*s ≥ 0.093). PTAs did not significantly differ between those with or without MCI (*p*s ≥ 0.292). Indeed, effect sizes comparing those with and without MCI for binaural processing (*ηp*^2^ = 0.029–0.034) were more than triple the effect sizes observed for PTA (right ear, *ηp*^2^ = 0.001; left ear, *ηp*^2^ = 0.004). Finally, Tuwaig et al. [30] found that DSI was significantly related to the following neurostructural and cerebrospinal fluid (CSF)-based biomarkers of early cognitive decline: cortex volumes, cortical thickness, and CSF tau levels. The aforementioned studies informed the selection of specific measures for the present study and underscore that the connection between binaural processing and cognition warrants further investigation.

Several theories have been suggested to explain the mechanisms that may link hearing and cognition [6,31,32,33], many of which were summarized by Uchida et al. [34] and, more recently, Bisogno et al. [35]. The cognitive load hypothesis suggests that the listening effort caused by hearing loss leads to cognitive reserve depletion, which in turn leads to cognitive decline [36,37]. The common cause hypothesis proposes that there may be a common mediating factor that is causing both hearing loss and dementia. For example, cardiovascular disease is a known risk factor for dementia [5] and a poor vascular supply to the cochlea can lead to hearing loss [1]. The cascade hypothesis proposes that the lack of auditory stimulation to the brain that results from hearing loss can lead to neuropathological alterations, such as cortical atrophy, thus leading to cognitive decline. Finally, the overdiagnosis hypothesis proposes that hearing loss, not cognitive decline, leads to poorer performance on cognitive assessments, which require the clinician to provide verbal instructions to the patient. While no single theory can sufficiently explain the relationship between hearing loss and cognition [35], individuals with hearing loss may be good candidates for targeted interventions designed to maintain or improve cognition.

While a large body of research concludes that there is a relationship between cognition and hearing, it remains unclear which aspects of each are the most significant contributors to this relationship. A prominent issue in the literature is the use of varied, inconsistent measures across studies. Several studies have called for future research to include more specific auditory tests or different tests of cognition [25,26,31,38,39]. The present study is unique in that the predictive value of peripheral (PTA4) and central auditory (DSI) measures is determined for a large array of cognitive domains measured using two tests of cognition (CBB with additional tests, MoCA with index scores). The overarching goal of this work is to elucidate the nature of the relationship between measures of hearing and cognition. Of paramount interest is whether PTA4 and/or DSI are predictive of performance on tests of cognition (MoCA, CBB). Based on previous literature, we hypothesize that: (a) if there is a relationship between hearing and cognition, greater hearing loss is expected to be associated with poorer cognition, and (b) DSI will be a better predictor of poor scores on cognitive measures when compared to the predictive power of PTA4.

## 2. Materials and Methods

### 2.1. Participants

The participants in this study are part of a 10-year longitudinal study, Indicators of Cognitive Change (ICC). To be included, participants must have been at least 60 years old at the baseline visit and had no reported diagnosis of dementia, neurodegenerative disease, or neurological injury. Participants were recruited by posting fliers digitally on research recruiting websites (e.g., VolunteerMatch) as well as physical postings in public areas, such as on the University of South Florida Tampa campus. Additionally, previous study participants who had agreed to be notified about new research opportunities were contacted and asked to participate. Hearing and cognitive data from 99 participants who completed baseline visits for the ICC study were included in the present study. The final sample was 77% female and primarily White (98%). Average participant age was 73 years, ranging from 60 to 92 years. Most study participants had a college degree. Demographic information is summarized in Table 1.

### 2.2. Measures

Cognitive measures were the CBB with additional tests (i.e., Groton Maze and International Shopping List) and the MoCA with index scores. The CBB is reliably sensitive to cognitive decline [40,41] and the MoCA is effective, reliable, and used clinically to detect cognitive impairment [42]. As for hearing measures, a standard audiometric evaluation [43] was performed and a four-frequency PTA (average thresholds at 500, 1000, 2000, and 4000 Hz) was calculated. DSI was used to assess auditory processing as it has been linked to several domains of cognition in the literature [20,28,29].

#### 2.2.1. Montreal Cognitive Assessment (MoCA)

The MoCA has been validated, is broadly utilized in clinical practice and research, and has demonstrated effectiveness as a screening tool for cognitive impairment [42,44]. The MoCA takes approximately 10 min to complete. The total possible score indicative of global cognition is 30 (with an additional one point possible for individuals with 12 years of education or less); a score of above 25 is generally considered within normal range. However, a growing literature proposes alternative cut-offs based on demographic variables including race, ethnicity, and years of education [45,46,47,48,49]. For the purposes of the present study, the MoCA global (i.e., total) score was treated as a continuous variable; participants were not grouped into cognitively “normal” or “abnormal” categories. The cognitive domains assessed are visuospatial and executive functioning, naming, attention, language, abstraction, delayed recall, and orientation. The sub-domain scores that may be extracted from the MoCA are memory (MIS), executive functioning (EIS), attention (AIS), language (LIS), visuospatial function (VIS), and orientation (OIS) index scores. Each of these sub-domains are calculated by summing scores of specific tasks as shown below. Note that the OIS was not included in the analyses as no prior literature has suggested a link between hearing and orientation. Detailed information including MoCA administration and scoring instructions can be accessed at mocacognition.com/paper/ (accessed on 24 August 2026).

- Memory (MIS): Number of words recalled in free delayed recall, category-cued recall, and multiple choice-cued recall, multiplied ×3, 2, and 1, respectively.
- Executive Functioning (EIS): summed points for trail making, clock drawing, digit span forward and backward, letter A tapping, serial 7 subtraction, letter fluency, and abstraction.
- Attention (AIS): summed points for digit span forward and backward, letter A tapping, serial 7 subtraction, sentence repetition, and words recalled in both immediate recall trials.
- Language (LIS): summed points for naming, sentence repetition, and letter fluency.
- Visuospatial Function (VIS): summed points for cube copy, clock drawing, and naming.
- Orientation (OIS; Not included): summed points for Date, Month, Year, Day, Place, and City.

The MoCA (MoCA Full, paper version 8.1) was administered to participants by trained, certified clinical research associates in strict accordance with administration, scoring, and interpretation guidelines provided by MoCA Cognition (https://mocacognition.com/training-certification/, accessed on 24 August 2026). The testing room was quiet and free from distraction. Following initial administration and scoring of the MoCA (at baseline and follow-up visits), tests were rescored by a different trained, certified research associate to optimize data quality and scoring accuracy. Discrepancies, although infrequent, were brought to the laboratory primary investigators for adjudication.

#### 2.2.2. Cogstate Brief Battery (CBB)

The CBB is an easy-to-use, computer-based cognitive screening tool for cognitively normal older adults and older adults with mild cognitive impairment [50,51,52]. The CBB was administered via desktop computer and consists of four tasks designed to assess verbal memory, reasoning, spatial memory, problem-solving, speed of processing, psychomotor function, attention, verbal learning, and working memory. Two additional CBB tasks were added to further assess learning, recall, and executive functioning for a total of six tasks (see list below). The CBB tasks were specifically designed to ensure test reliability and validity regardless of participants’ ethnicities, socio-economic backgrounds, or level of education. Multiple studies have documented high (*r* > 0.70) test–retest reliability of the CBB, while others have demonstrated strong correlations (*r* = 0.49 to 0.83) between the CBB tasks and formal neuropsychological tests [41,52,53,54]. The CBB was originally intended to be compared with each subject acting as their own standardized norm as a measure of change, rather than a measure of function. Thus, this test cannot provide a cut-off value for what is considered abnormal cognitive function but is still a valuable tool for measuring multiple aspects of cognition. Detailed descriptions and demonstrations of the tasks may be found at https://www.cogstate.com/digital-cognitive-assessment/ (accessed on 20 July 2026).

The instructions for each CBB task were displayed onscreen; clinical research associates read the instructions aloud to participants using a script provided in the CBB testing manual. Each CBB task included a brief set of practice trials to ensure that participants understood how to complete the task. The first four CBB tasks utilized playing cards that appeared one at a time in the center of the screen.

Participants were instructed to press either a “Yes” or “No” key in response to each trial in the task:

- Detection Task (2 min; 35 trials) is a simple reaction time task that measures psychomotor functions and speed of processing. In each trial, participants were shown a face-down card and instructed to press “Yes” as soon as the card turned face-up.
- Identification Task (2–3 min; 30 trials) is a choice reaction time task that measures visual attention and vigilance. In each trial, a face-down card would turn face-up, revealing a red or black design. Participants were instructed to press “Yes” if the card was red or “No” if the card was black.
- One-Card Learning Task (5 min; 42 trials) is a measure of visual learning and memory. In each trial, participants were shown one card at a time and were instructed to press “Yes” if they had previously seen the card in the task, or “No” if they had not previously seen the card. The CBB randomly selects some cards from the deck to repeat during the task and mixes them with novel cards.
- One-Card Back Task (2 min; 30 trials) is a measure of attention and working memory. In each trial, participants were shown one card at a time and were instructed to press “Yes” if the card was the same as the one presented immediately before it, or “No” if it was not the same.

Additional tasks:

- Groton Maze Learning Task (10 min) utilizes a maze learning paradigm used to measure executive function, spatial working memory, delayed recall, learning efficiency, and error monitoring. Participants were required to follow a hidden pathway in a 10 × 10 grid shown onscreen by clicking individual tiles in the grid using the computer mouse. After each click, a message informed the participants whether their move was correct, or if they needed to return to the previous tile and try again. Participants completed a total of five trials using the same hidden pathway [55].
- International Shopping List Task (7 min) utilizes a word list learning paradigm to measure verbal learning and delayed recall. Research assistants read aloud a list of 12 common grocery items, at a rate of one word per second. Participants were asked to recall as many items as possible. Participants completed a total of four trials using the same list.

#### 2.2.3. Audiometry

Each participant received a standard audiometric evaluation [43] with calibrated equipment and insert earphones. This evaluation included pure-tone audiometry as well as speech recognition thresholds (SRTs; using spondees). Testing was conducted via insert earphones for each ear at the following frequencies: 250 Hz, 500 Hz, 1000 Hz, 2000 Hz, 4000 Hz, and 8000 Hz. PTA4 (average thresholds for 500, 1000, 2000, and 4000 Hz) was calculated for each ear. The left and right ear PTA4s were combined for statistical analyses.

#### 2.2.4. Dichotic Sentence Identification (DSI)

The DSI assesses dichotic sentence understanding [56], has been shown to be a valid measure of binaural auditory processing [56]. The DSI has moderate reliability among adults (*rs* = 0.42–0.55) [57] with some indication that reliability may be poorer among older adults [58]. The stimuli were nonsense sentences (e.g., “Go change your car color is red”) and audibility was ensured by setting the level of presentation for each ear individually at 50 dB SL (re: SRT) via insert earphones. Two sentences were presented at the same time, with one sentence delivered to each ear. The talker was male and approximately eight seconds of silence separated each of the trials. Participants selected the two sentences they heard from a list of six possible nonsense sentences. Thirty trials were completed. The scores obtained were the percent of correct sentences from the right ear, left ear, and total out of the thirty trials. For this study, the total percent correct was used.

### 2.3. Procedures

The study visit included informed consent documentation, a demographic and eligibility form, the MoCA, the CBB (with the addition of the Groton Maze and International Shopping List tasks), the audiometry evaluation, and the DSI. All data collected were stored in a USF REDCap database. All data were scored and rescored by different data collectors for quality control and subsequently rechecked in REDCap by another set of data collectors before being marked as a complete data sample for that visit. Descriptive analyses were conducted via SPSS (IBM SPSS Statistics version 31.0.0.0 (117)), and other analyses were conducted with R (version 4.5.1) [59]. Pearson’s *r* and multivariate analyses were used to investigate the nature of the relationships between each of the variables (MoCA, CBB, combined PTA, DSI total).

## 3. Results

Mean performance for hearing and cognitive measures is summarized in Table 2. The PTA4 ranged from 8 dB HL to 66 dB HL, and DSI scores ranged from 0 to 30. Overall, PTA and DSI scores were weakly correlated (*r* = −0.35). MoCA total scores ranged from 20 to 30.

### 3.1. MANOVA

A type 2 Multivariate Analysis of Variance (MANOVA) with Pillai’s Trace was conducted to determine how well PTA4 and DSI predict performance on the MoCA and CBB. For the MANOVA, three tests were conducted: combined MoCA-CBB scores, MoCA-only scores, and CBB-only scores. A Bonferroni correction was used to reduce the possibility of a Type 1 error (α = 0.017) arising from the method of analyses. The results of these analyses can be found in Table 3. Most notably, the DSI was the primary predictor for both the combined MoCA-CBB analysis and the CBB-only scores. Neither of the tests were significant predictors of MoCA-only scores.

### 3.2. Correlations

To further explore the relationship between hearing and cognitive measures, Pearson’s product moment correlations were calculated between all hearing variables and all cognitive variables. The results are summarized in Table 4. The strongest correlation, a moderate negative correlation (*r* = −0.40), was found between the Groton Maze and DSI. The DSI also demonstrated a greater number (8) of weaker, but still meaningful, correlations with sub-scores on both cognitive tests. The PTA4 scores were weakly related to four cognitive variables, but no moderately strong correlations were observed.

Finally, it was observed that the MoCA and CBB did not display similar relationships with the auditory measures. This prompted a post-hoc examination of the correlations between cognitive measures themselves. Table 5 shows correlations among MoCA and CBB variables in which three moderately strong relationships (Attention Index Score and One-Card Back; Attention Index Score and International Shopping List; MoCA total and International Shopping List) and 15 weak correlations were observed; the remainder (18) were uncorrelated.

## 4. Discussion

This study is the first to investigate the relationships between both peripheral and central auditory function and multiple domains of cognition from two commonly used cognitive assessment tools (CBB and MoCA). Hearing loss and cognitive impairment (due to AD and related causes) are costly and prevalent conditions among older adults. The results suggest that the ability to separate binaurally presented auditory information is strongly related to specific aspects of cognition, with the pattern of results varying across the cognitive assessment tool used.

The DSI was the only significant predictor across cognitive tests. Additionally, the DSI and the Groton Maze task from the CBB exhibited a moderate negative relationship. Since Groton Maze tasks are reverse-coded, this relationship means that DSI and Groton Maze have a direct correlation. This finding connects dichotic sentence processing with several cognitive domains evaluated by the Groton Maze, including executive function, spatial working memory, learning efficiency, delayed recall, and error monitoring. This result should be considered with the significant but weaker relationships between DSI and measures of attention (CBB Identification), learning and memory (CBB One-card Learning, International Shopping List), attention and memory (One-card Back), global cognition (MoCA total score), executive function (MoCA EIS), attention (MoCA AIS), and visuospatial function (MoCA VIS) and the fact that relationships between DSI and speed of processing (CBB Detection), memory (MoCA MIS), and language (MoCA LIS) were not found. This collection of results suggests that binaural processing is strongly related to the broad cognitive domains of executive function and attention but less so to memory.

In contrast, PTA4 showed fewer, weak correlations, the strongest of which were learning and memory (International Shopping List, One-Card Learning) and attention (MoCA AIS). Based on these results, our hypotheses were supported. Greater hearing loss is associated with poorer performance on some cognitive tests but not others, and DSI (auditory processing) is a better predictor of poor cognitive test performance when compared to the predictive power of PTAs (peripheral hearing).

The pattern of weak correlations between MoCA and CBB measures is not easy to explain. Even for tasks that seem very similar in nature, the cognitive measures appear to be capturing different aspects of cognition. For example, performance on the MoCA memory sub-test (MIS) and several of the CBB measures that require memory (One-Card Learning, One-Card Back, Groton Maze) were not related. This is likely at the root of the mixed findings currently present in the literature [26,27]. When cognition is measured broadly (e.g., MoCA total score) or defined using a single measure of a single domain (e.g., memory), relationships with auditory function may not be apparent. Similarly, when “hearing” is measured using a single self-report measure or PTA, relationships with cognitive function may be weak. Other authors have also reported on the ambiguity of the relationship between cognition and hearing/auditory processing due to the variety of measures and mixed results [60,61].

A final point to be made in the interpretation of these results is that numerous studies in this area have investigated associations between hearing loss and later stages of dementia, with fewer studies examining earlier stages in the disease process. As a result, there is a lack of research on the hearing/cognition relationships across the broad cognitive health continuum, which would include normative age-related and preclinical (asymptomatic) processes [62,63]. There is a current need for studies that include older adults with a wide range of both auditory and cognitive abilities. While the current study did include a fairly wide range of abilities, the average level of impairment was relatively mild. In demographic terms, our sample of participants was quite homogenous, which limits the generalizability of the present findings. Sample homogeneity is not uncommon in academic institution-based research studies—particularly among those examining brain health and cognition in older adults. In this area of research, it is typical for studies to have a greater representation of older adults who are healthier (cognitively and physically), ambulatory, more physically active, and more highly educated [64,65,66]. It is clear that future studies should make concerted efforts to recruit participants who are more broadly representative of the older adult population. To that aim, the National Institutes of Health and the National Institute on Aging have developed the Alzheimer’s and Dementia Outreach, Recruitment, and Engagement (ADORE) tool which includes a broad collection of resources centered on the recruitment and retention of older adult research participants—particularly those from historically underrepresented groups. Strategies for increasing sample heterogeneity focus on the minimization of common barriers to participation using multi-modal, community-based approaches including but not limited to strengthening community partnerships and trust (e.g., USF Workgroup Enhancing Community Advocacy and Research Engagement; WE-CARE), engaging older adults through community educational presentations, strategic direct mailings, complimentary health outreach initiatives (e.g., hearing and cognitive screenings), and providing travel to and from study visits and other practical accommodations [67]. Partnerships with academic and community-based memory disorder clinics can bolster variability in cognitive performance among study participants. Another limitation of the present study is lack of normative data for some of the tests used. As the DSI does not have established values for what is considered impaired for older adults in each ear, normative data would be needed prior to any clinical implementation of these findings. Additionally, the CBB was originally intended for intrapersonal cognitive monitoring over time with each participant acting as their own norm or baseline, and this should be considered if it is to be integrated into a clinical test battery successfully.

Further research in this area could lead to the development of clinical versions of some of the tests of interest in this study, such as a shorter Groton Maze task or an automated DSI test that could easily be completed by patients during appointments. As mentioned, several studies have shown that patients are willing to participate in preventative care for dementia, so it would be crucial to develop translational research on how to apply these findings to real-world clinical workflows. Preventative strategies (e.g., treating hearing loss in advance of cognitive symptom onset) that potentially reduce risk for this debilitating disease would be life-changing for many patients and their family members. Clinical trials investigating the utility of hearing interventions, such as hearing aids, for improving cognitive outcomes are needed. Longitudinal data illustrating how cognition changes over time in relation to hearing will be critical in furthering our understanding of the connection between hearing and cognition. Also useful would be studies integrating auditory measures with established biomarkers of dementia and neurodegenerative disease and research in individuals with MCI to determine whether specific auditory measures may contribute to earlier identification of individuals at increased risk of cognitive decline.

The results of this study have advanced our understanding of the specific domains of hearing and cognition that are related, thus informing theory and, ultimately, setting the stage for translation to clinical practice. These results may be interpreted as a call for closer collaboration among audiologists, primary care practitioners, neuropsychologists, geriatricians, and other allied health professionals to create interdisciplinary approaches to patient-centered care that may facilitate the identification and management of older adults susceptible to cognitive decline. The Gerontological Society of America’s (GSA) Kickstart, Assess, Evaluate, Refer (KAER) holistic framework [68] provides valuable guidance and tools for practitioners geared toward early detection, assessment, and collaborative brain health care well-suited to hectic clinic workflows.

It appears that the ability to separate and identify competing auditory information may be important for efficient and effective cognitive processing which provides support for the cognitive load hypothesis. This hypothesis is based on the premise that the central pool of cognitive resources is finite. Hearing loss requires an increase in listening effort (i.e., attentional and executive processes seated in the frontoparietal network [FPN] necessary for speech comprehension) to compensate for degraded peripheral auditory signals [36,69,70]]. Compensatory resources are recruited (i.e., upregulated) in frontoparietal brain regions (i.e., FPN) that facilitate attentional control and executive functions (e.g., inhibition)—crucial processes associated with auditory perception [69]. When these finite resources are allocated to the processing of basic incoming auditory signals, fewer resources remain for higher-level cognitive tasks such as working memory and encoding [37]. It is posited that, in the short-term, secondary task performance will deteriorate and, over time, sustained load will ultimately result in cognitive decline due to the depletion of cognitive reserve [37]. Deficits in binaural processing that occur with age may reduce one’s ability to process incoming information, thus depleting cognitive reserve and resulting in cognitive decline [33,34].

In terms of clinical practice, audiologists should be aware that subjective complaints of difficulty understanding competing auditory messages by patients should be noted, as this may be indicative of a cognitive issue that may influence hearing health outcomes. Such complaints, especially when observed along with episodes of confusion or memory impairment during scheduling or conducting appointments, may warrant use of a brief screening tool to help facilitate referral to the patient’s preferred health care provider for thorough assessment. Several efficient, validated cognitive screening measures (3- to 5-min administration) that can be easily integrated into busy clinical settings (e.g., Mini-Cog [71], 5-Cog [72]) are freely available in written and/or computer/tablet-based (Mini-Cog) formats. Increased awareness and utilization of brief cognitive screening measures among at-risk older adults will greatly contribute to early detection and treatment, when applicable, thereby contributing significantly to patients’ overall health, wellness, and well-being.

## Figures and Tables

**Table 1:** Participant description (*n* = 99).

	Mean (SD) or Percent
Age (years)	73.04 (6.87)
Education (years)	16.59 (1.7)
Race (%White)	98%
Ethnicity (%Non-Hispanic)	98%
Gender (%Male)	23%

**Table 2:** Hearing and Cognitive Measures (*n* = 99).

Measure		Mean (SD)
MoCA	Memory Index Score (MIS)	13.0 (2.44)
	Executive Function Index Score (EIS)	11.5 (1.34)
	Attention Index Score (AIS)	16.8 (1.48)
	Language Index Score (LIS)	5.4 (0.84)
	Visuospatial Index Score (VIS)	6.1 (0.97)
	Total Score	26.2 (2.7)
CBB	Detection	2.59 (0.08)
	Identification	2.75 (0.06)
	One-Card Learning	0.99 (0.11)
	One-Card Back	1.32 (0.20)
	Groton Maze	9.82 (6.16)
	International Shopping List	1.05 (0.27)
Hearing	DSI Total Score	22.3 (7.48)
	Combined PTA4 (dB HL)	28.4 (13.6)

**Table 3:** Type II MANOVA Tests: Pillai Test Statistic.

		DF	Test Stat	Approx F	Num DF	Den DF	Pr(>F)
Combined Cognitive Measures	Combined PTA4	1	0.13	1.10	12	85	0.373
	DSI Total	1	0.31	3.15	12	85	0.001 *
CBB	Combined PTA4	1	0.06	0.92	6	91	0.487
	DSI Total	1	0.28	6.04	6	91	2.37 × 10^−5^ *
MoCA	Combined PTA4	1	0.10	1.63	6	91	0.149
	DSI Total	1	0.11	1.81	6	91	0.106

* significant at the 0.017 level (2-tailed).

**Table 4:** Pearson’s *r* calculations comparing auditory and cognitive measures.

Variable		DSI Total	Combined PTA
CBB	Detection	−0.02	0.12
	Identification	−0.21 *	0.17
	One-Card Learning	0.37 *	−0.27 *
	One-Card Back	0.29 *	−0.20
	Groton Maze	−0.40 **	0.16
	International Shopping List	0.34 *	−0.25 *
MoCA	MIS	0.14	−0.08
	EIS	0.31 *	−0.07
	AIS	0.26 *	−0.25 *
	LIS	0.19	−0.12
	VIS	0.29 *	−0.21 *
	Total	0.32 *	−0.13

** moderately strong relationship. * weak relationship.

**Table 5:** Pearson’s *r* calculations comparing CBB and MoCA cognitive measures.

		MIS	EIS	AIS	LIS	VIS	Total
Detection	Pearson Correlation	0.09	0.05	0.08	−0.13	−0.15	0.03
Identification	Pearson Correlation	0.06	−0.02	−0.02	−0.28 *	−0.05	−0.06
One-card Learning	Pearson Correlation	0.19	0.29 *	0.34 *	0.16	0.27 *	0.37 *
One-card Back	Pearson Correlation	0.14	0.20 *	0.43 **	0.19	0.21 *	0.28 *
Groton Maze	Pearson Correlation	−0.28	−0.24 *	−0.16	0.04	−0.31 *	−0.28 *
International Shopping List	Pearson Correlation	0.36 *	0.30 *	0.45 **	0.26 *	0.22 *	0.48 **

** moderately strong relationship. * weak relationship.

## Data Availability

The data that support the findings of this study are available upon request to Principal Investigator Jennifer Jones Lister by e-mailing jlister@usf.edu and with completion of a data use agreement.

## Abbreviations

The following abbreviations are used in this manuscript:

PTA	pure-tone average
PTA4	four-frequency pure-tone average
DSI	Dichotic Sentence Identification
MoCA	Montreal Cognitive Assessment
CBB	Cogstate Brief Battery
MCI	mild cognitive impairment
MIS	Memory Index Score
EIS	Executive Function Index Score
AIS	Attention Index Score
LIS	Language Index Score
VIS	Visuospatial Index Score
USA	United States of America
USF	University of South Florida
AD	Alzheimer’s disease
ADRD	Alzheimer’s disease and related dementias
dB HL	decibels hearing level
ADORE	Alzheimer’s and Dementia Outreach, Recruitment, and Engagement
WE-CARE	Workgroup Enhancing Community Advocacy and Research Engagement
GSA	Gerontological Society of America
KAER	Kickstart, Assess, Evaluate, Refer holistic framework
FPN	frontoparietal network
